# Controlling *Listeria monocytogenes* Scott A on Surfaces of Fully Cooked Turkey Deli Product Using Organic Acid-Containing Marinades as Postlethality Dips

**DOI:** 10.1155/2015/157026

**Published:** 2015-06-25

**Authors:** Gerardo Casco, Jennifer L. Johnson, T. Matthew Taylor, Carlos N. Gaytán, Mindy M. Brashears, Christine Z. Alvarado

**Affiliations:** ^1^Department of Poultry Science, Texas A&M University, College Station, TX 77843-2472, USA; ^2^Department of Animal and Food Sciences, Texas Tech University, Lubbock, TX 79409-2141, USA; ^3^Department of Animal Science, Texas A&M University, College Station, TX 77843-2471, USA; ^4^Colegio de Postgraduados Campus Cordoba, 94946 Cordoba, VER, Mexico

## Abstract

This study evaluated the efficacy of organic acids applied singly or in combination as postlethality dips to sliced uncured turkey deli loaves to inhibit the growth of *Listeria monocytogenes* (Lm) Scott A. Treatments consisted of sodium lactate (SL; 3.6%), potassium lactate (PL; 3.6%), sodium citrate (SC; 0.75%), a combination of SL and sodium diacetate (SDA; 0.25%), and a combination of SL/PL/SDA, alongside appropriate negative and positive controls. Products were inoculated with 10^4^–10^5^ CFU/mL streptomycin-resistant (1500 *μ*g/mL) Lm Scott A prior to treatment. Products were then stored at ~4°C and sampled at 0, 7, 14, 21, 28, 42, and 56 d. The SL/SDA combination applied to turkey slices extended the lag phase through 21 days of refrigerated storage. Numbers of Lm Scott A rose by 0.7 log_10_ CFU/g through the 56 d storage period. The application of the SL/PL/SDA treatment to turkey product surfaces extended the lag phase through 42 d, with pathogen numbers declining after 21 d. Combination organic acid dips prolonged the lag phase for 2 to 6 wk on turkey product surfaces and can be useful as antimicrobial agents for Lm control on postlethality exposed sliced deli products.

## 1. Introduction

The US Centers for Disease Control and Prevention (CDC; Atlanta, GA) [1] reported 1651 cases of human foodborne listeriosis from* Listeria monocytogenes* (Lm) which occurred from 2009 to 2011, with a case-fatality rate of 21%. Nevertheless, the US Department of Agriculture Food Safety and Inspection Service (USDA-FSIS) has reported a decline in the prevalence of Lm in ready-to-eat (RTE) meat and poultry products (including deli products) collected through its sampling program, from 4.61% in 1990 to 0.27% in 2011 [2]. The USDA has also recently provided guidance for the control of Lm growth on RTE meats, including the proper application of antimicrobial agents (AAs) in the manufacturing of deli products [3]. Antimicrobial hurdles consist of a combination of several processes and/or interventions that result in a growth-limiting impact on pathogenic bacteria. Commercial AAs may contribute to the hurdles by reducing the water activity (*a*_*w*_) of foods, lowering product pH, REDOX potential modulation, or combination of these and other processes [3–5].

Organic acids and their salts applied to RTE deli products either in the product formula or to the product's surface postlethality and are known to possess Lm growth-inhibiting capabilities. Islam et al. [6] tested the ability of organic acid marinades applied to turkey frankfurters as postcook dips to reduce Lm growth, reporting reductions of 1.0 to 2.0 log_10_-cycles compared to nontreated controls. Carroll et al. [7] evaluated the addition of organic acid marinades to turkey deli loaf formulae and found the Lm lag phase was extended through 56 days on slices treated with a 3.0% sodium lactate and 0.25% sodium diacetate combination. Porto et al. [8] concluded that incorporation of potassium lactate (PL) into vacuum-sealed frankfurters stored at 4°C and 10°C provided listeriostatic effects, extending the lag phase for the duration of the study (90 and 60 days, resp.) compared to a frankfurter without PL. Nuñez De Gonzalez et al. [9] reported an overall growth inhibition of ≤2.0 log_10 _CFU/frank in Lm counts on beef frankfurters formulated without or with 3.3% PL, and surface dip-treated with potassium lactate (3.3%) or lactic acid (3.4%) over 12 wk refrigerated storage. Barmpalia et al. [10] found that growth was inhibited in frankfurters formulated with lactate and diacetate and then treated with lactic or acetic acid postcook dips.

Antilisterial properties of differing organic acids and their salts can be affected by processing methodology and product formulae [8]. In addition, despite the establishment of the USDA's “zero tolerance” policy for Lm on RTE foods and consequent decline in the prevalence of the pathogen on RTE products,* L. monocytogenes* remains a persistent contaminant on RTE deli products with high severity [2, 9]. The objective of this study was to evaluate the effect on an isolate of Lm Scott A growth caused by sodium lactate (SL), potassium lactate (PL), sodium citrate (SC), and sodium diacetate (SDA), applied individually or in combination (SL/SDA, SL/PL/SDA) as postcook dips to inoculated turkey deli loaf slices.

## 2. Materials and Methods

### 2.1. Bacterial Microorganism and Handling Procedures

*L. monocytogenes* Scott A, obtained from the Auburn University (Auburn, AL) frozen culture collection, was stored in 1.5 mL microcentrifuge tubes (VWR Int., West Chester, PA, USA) at −80°C prior to revival. To revive the organism, 1.0 mL of frozen culture was pipetted into 9.0 mL sterile brain heart infusion (BHI) broth (Oxoid, Basingstoke/Hampshire, England) and incubated overnight at 37°C. Turbid cultures were transferred to virgin BHI medium and subculturing was repeated in identical fashion. Five 1 : 10 serial dilutions were prepared into tubes containing 9.0 mL sterile phosphate buffered saline (PBS) (Pierce Perbio Thermo Scientific, Bonn, Germany). To determine the growth of Lm Scott A following subculturing, dilutions were plated in duplicate on 15 × 100 mm Petri plates containing BHI agar (Difco, Lawrence, KS, USA), followed by overnight incubation at 37°C. The incubated plates were enumerated with photographic ColorCount recognition technology using the *Q* count instrument (Spiral Biotech, Norwood, MA, USA).

Streptomycin resistance (Strep^*R*^) was induced through exposure of the organism to antibiotic in medium.* L. monocytogenes* Scott A cells were gradually exposed to increasing concentrations of streptomycin in BHI broth and BHI agar according to previous procedures [11]. A stock solution was prepared by mixing streptomycin sulfate (Sigma-Aldrich Co., St. Louis, MO, USA) in sterile distilled water with stirring and gentle heating when necessary. The solution was filter sterilized into 250 mL bottles (Pyrex, Lowell, MA, USA) using a 60 mL syringe filter (0.2 *μ*m pore diameter) (VWR Int.). This then was added to sterilized medium in a laminar flow hood to the desired exposure concentration. Pending survival at the initial exposure concentrations (0.2, 1.6, 6.4, and 12.8 *μ*g/mL) antibiotic concentration applied in broth and agar was systematically doubled to increase Lm resistance, until a maximal resistance of 1,500 *μ*g/mL was obtained. Desired cultures were subsequently frozen in sterile 1.5 mL microcentrifuge tubes and stored at −80°C until required for use. The strains were initially incubated in 10 mL of BHI broth with 10% glycerol solution at 37°C [11]. From this, 300 *μ*L was added to a 700 *μ*L volume of glycerol in microcentrifuge tubes and vortexed prior to freezing. A sample of each strain was subsequently thawed and cultured to determine bacterial survival following the procedure.

Strep^*R*^ cultures were grown on BHI slants and submitted for identification by the USDA-Agricultural Research Service (ARS; Athens, GA, USA). Cultures were streaked on modified Oxford's (MOX) agar (Remel, Lenexa, KS, USA) for cultivation of characteristic small-black colonies. A sterile loop was used to collect 5 to 10 colonies for *β*-hemolysis identification on horse blood overlay plates. At least two colonies were collected from hemolysis-positive plates for confirmatory testing including Gram-stain, CAMP test, rhamnose utilization, catalase reaction, and tumbling motility under wet mount [12].

### 2.2. Turkey Deli Loaf Preparation

Preparation of a turkey loaf product followed industry standard procedures. Turkey lobe meat (Patuxent Farms, Columbia, MD, USA) was ground (20%) using a Waring grinder (Waring Products, Inc., Torrington, CT, USA) while the remaining meat (80%) was hand-sectioned into 1.0 in^3^ cubes. Deli product formulation incorporated noniodized refined salt (1.5%; Morton, Chicago, IL, USA) and sodium tripolyphosphate (STP; 0.45%, Innophos, Cranbury, NJ, USA) in a 20% marinade (7 : 3 water : ice ratio) of the final batch weight. Marinade was prepared using a handheld biohomogenizer (ESGE m13333-1281-O, Bartlesville, OK, USA) until all ingredients were dissolved and incorporated through vacuum tumbling (Hollymatic Corp., Countryside, IL, USA) for 1 h at 25 mm Hg, 14 rpm, at 4°C. This was followed by equilibration at 4°C for 3 h. All meat used was maintained at 4°C through transport and processing. The marinated turkey batter was manually stuffed into 10.16 cm diameter fibrous cellulose casing (EZ Peel fibrous casing, Viskase Companies, Inc., Willowbrook, IL, USA) by hand crank manual stuffer (Koch, Kansas City, MO, USA); each loaf was approximately 63.5 cm in length. Loaves were trucked into a single truck smokehouse (Alkar, Lodi, WI, USA) and cooked according to a stepped processing schedule (Table 1). Loaves were then removed and chilled overnight to an internal temperature of ~4°C. A turkey loaf was hand peeled and sliced into ~2 mm slices (9512 12′′ Max Manual Meat Slicer, Univex Corp., Salem, NH); the blade was calibrated by caliper and sanitized with 70% (v/v) ethanol before being used again (Marathon Watch Co., Ontario, Canada). Ten slices were individually weighed and averaged to obtain an approximate slice weight to be used in later calculations. Slices were collected onto a table lined with sterile foil prior to inoculation and treatment with different postcook antimicrobial dips.

### 2.3. Deli Meat Slice Inoculation Procedures

The Lm Scott A culture was passed at least three times before inoculation to allow for revival as described above. The Lm Scott A stock was decimally diluted from inoculated BHI broth into the necessary sterile test tube containing 9.0 mL sterile PBS to generate countable plates (25–250 CFU). Each dilution was spread in duplicate on 15 × 100 mm Petri plates containing BHI agar and incubated overnight at 37°C. The incubated plates were enumerated with a photographic ColorCount recognition technology using the *Q* count instrument (Spiral Biotech). The working stock solution was prepared to target 10^5^ CFU/g on turkey slices. Each slice (*n* = 3) was inoculated with 0.1 mL stock culture using 10 *μ*L aliquots. Disposable, sterile 10 *μ*L volume inoculating loops (VWR Int.) were used to spread inoculum across the surfaces of meat slices. This was performed on a single side of the slice. Inoculated slices were set aside for 2 min to allow for bacterial attachment. Transfer of slices to segregated stations was conducted using ethanol/flame-sterilized forceps.

### 2.4. Organic Acid Dip Immersion Treatments

Organic acid treatments applied individually and in combination to the Lm Scott A-inoculated slices were (1) sodium lactate (SL; 3.6%, Fisher Scientific, Pittsburgh, PA); (2) potassium lactate (PL; 3.6%, City Chemicals, West Haven, CT); (3) sodium citrate (SC; 0.75%, Fisher Scientific); (4) sodium lactate (SL; 3.6%) + sodium diacetate (SDA; 0.25%, Spectrum Chemical Manufacturing Corp., Gardena, CA); (5) SL (3.6%) + PL (3.6%) + SDA (0.25%); (6) positive control (LM-inoculated slices dipped in sterile distilled water); and (7) negative control (noninoculated slices dipped in sterile distilled water). Slices were dipped in 100 mL freshly prepared solution for 1.0 min and removed with sterile forceps for drying on sterile foil-lined drying racks. Excess dip fluid was allowed to dry for at least 10 min with intermediate flipping of the slices. Dry slices were packaged in groups of three, by treatment, using 24 oz. Whirlpak bags (Nasco, Fort Atkinson, WI) and stored at ~4°C until their appropriate sampling times were reached. Slices were stored aerobically due to nonavailability of biosafety level 2 (BSL2)-compliant vacuum sealing technology in the research pilot plant laboratory. Three slices were allocated to each of 7 time points per each of the 5 treatments, totaling 105 slices per replicate; two additional slices per time point were allotted for a positive and negative control, providing an additional 28 slices.

### 2.5. Microbiological Testing of Treated Slices

Packaged slices were removed from refrigerated storage for sampling on days 0, 7, 14, 21, 28, 42, and 56. Sterile forceps were used to divide the slices into individual filtered stomacher bags. Each bag was filled with 50 mL sterile PBS and homogenized using a Stomacher 400 Circulator (Seward, West Sussex, UK) for 2 min at 245 rpm. Decimal dilutions were prepared prior to inoculation onto streptomycin-loaded (1,500 *μ*g/mL) BHI agar-containing Petri plates. Samples were incubated for 48 h at 35°C prior to colony enumeration. Duplicate plate counts were taken, subjected to log_10_-transformation, and averaged. The average slice weight was considered with 50 mL PBS added to create the first dilution.

### 2.6. Statistical Analysis Procedure

The log_10_/g average plate counts from each of three inoculated slices treated in identical fashion with one of the 7 treatments at each of the seven sample points (0, 7, 14, 21, 28, 42, and 56 days) over three identically completed replicates (*n* = 3) were subjected to statistical analysis using the PROC GLM procedure of SAS v11.0 (SAS Institute, Inc., Cary, NC). Separation of statistically differing means was carried out within a sampling period across treatments using Duncan's Multiple Ranges Test at *α* = 0.05.

## 3. Results and Discussion

The potential for Lm Scott A survival on food processing machinery, environmental niches, and fixtures from processing facilities is of great concern as cross-contamination of deli products can occur during postlethality handling [13]. The use of postlethality antimicrobial dips could provide a barrier in the control for* L. monocytogenes* growth inhibition.

**Figure 1:**
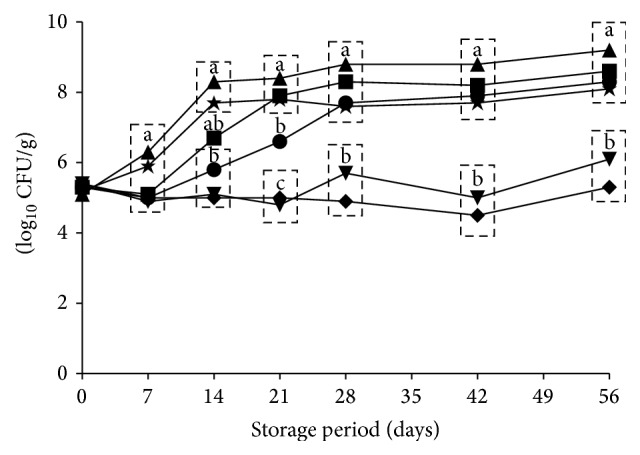
Inhibition of streptomycin-resistant (1500 *μ*g/mL)* Listeria monocytogenes* Scott A on fully cooked uncured deli turkey slices treated by organic acid postcook dips applied singly or in combination, packaged and stored aerobically at 4°C. Symbols depict means from triplicate identical replications (*n* = 3), pooled by treatment: 3.6% sodium lactate (●), 3.6% potassium lactate (■), 0.75% sodium citrate (▲), 3.6% sodium lactate + 0.25% sodium diacetate (▼), 3.6% sodium lactate + 3.6% potassium lactate + 0.25% sodium diacetate (*◆*), and inoculated, nontreated control (★). Sliced product was treated by 1 min dip exposure to antimicrobial dips and then aseptically handled for enumeration of* L. monocytogenes* on streptomycin (1500 *μ*g/mL) supplemented brain heart infusion (BHI) agar following 24 hr incubation of inoculated Petri plates at 37°C. Symbols enclosed within dashed boxes do not differ at *p* < 0.05, while sampling day-specific pooled treatment means bearing differing letters (a, b, c) differ at *p* < 0.05.

The inhibition of Lm Scott A on uncured turkey loaf slices by postcook organic acid dips is depicted in Figure 1. Combination treatments of SL/SDA and SL/PL/SDA suppressed Lm Scott A cell growth postprocessing (21 and 42 d, resp.) on turkey deli loaf slices compared to the other treatments on those sampling days (*p* < 0.05). SL and PL treatments (*p* < 0.05) inhibited growth at day 7 when compared to the pathogen-inoculated positive control and the SC-treated slices. However, at 28 days' refrigerated storage no differences were observed between the control and the SC treatment. Throughout the experimental period, Lm Scott A counts on SC-treated product did not differ from the inoculated positive control on any sampling day (*p* ≥ 0.05) (Figure 1). Overall, combination of SL (3.6%) plus SDA (0.25%) or SL (3.6%) + PL (3.6%) + SDA (0.25%) demonstrated inhibition of Lm Scott A growth on post-dipping refrigerated turkey deli slices over 56 days' storage.

Antimicrobial activity of weak organic acids, including lactate, citrate, and acetate, is generally attributed to penetration of the undissociated acid molecule through the cell membrane, with resulting acidification of the cytoplasmic space and activation of active processes to reestablish internal pH homeostasis [14]. Weak organic acids such as lactic and citric acid are favored for intracellular permeability due to inherent hydrophobicity and the resulting interactions with the cellular lipid bilayer. This may result in cell damage, death, or exhaustion due to expenditure of energy and resources to exclude the acid. Additionally, lactic acid has been reported to permeabilize the outer membrane of some Gram-negative organisms, and multiple weak organic acids are reported to exert antimicrobial activity in the form of DNA degradation or ribosomal attack via the dissociated acid anion once the cytoplasm is reached [15, 16].

Extensive published research supports organic acids and their salts as effective AAs in poultry deli product formulae. However, knowledge of these AAs as postcook dip solution ingredients and their effects on Lm in poultry deli meat slices is not as extensive [8, 13, 17–20]. Lianou et al. [21] simulated in-plant cross-contamination and found that combinations of commercial lactate (1.5%) and diacetate (0.05%) formulated into uncured turkey breast reduced Lm growth rates compared to untreated turkey breast over 12 d of storage at 7°C. Carroll et al. [7] found a combination treatment of 3% SL and 0.75% SDA applied to inoculated, vacuum-packaged turkey deli slices extended the lag phase of Lm through 63 d of storage at 4°C. The combined application of 2.0% SL + 0.25% SDA via edible coatings on fully cooked turkey breast stored at freezing resulted in enhanced reduction in Lm numbers versus coatings formulated with SL or SDA alone [22]. Collectively, combined application of lactate and diacetate solutions produces a greater antilisterial effect on fully cooked poultry deli products subject to Alternatives 1 or 2a of Title 9 of the US Code of Federal Regulations (CFR) §430. Samelis et al. [23] noted that combination treatments of 0.25% SL with 0.25% SA, SDA, or glucono-*δ*-lactone applied to frankfurters extended the Lm lag phase throughout 120 d at refrigerated storage. Similarly, Palumbo and Williams [24] reported that inoculated, vacuum packaged frankfurters treated with a combination of 2.5% citric acid and 2.5% acetic acid dip suppressed Lm growth over 85 d at refrigerated (5°C) storage.

*L. monocytogenes* isolates are reported to vary in their sensitivity to antimicrobial exposure, including exposure to organic acids. Brandt et al. [25] reported Lm Scott A exhibited greatest sensitivity to nisin with a minimum inhibitory concentration approximately half that of other isolates tested. Similarly, Cheng et al. [26] reported that Lm M7, an avirulent isolate, demonstrated significantly slower growth and in some instances failed to grow and replicate in liquid medium infused with acetate or lactate to pH 5.5. Authors hypothesized that deletion of a sigma factor in the avirulent M7 Lm isolate was responsible for loss of pH homeostasis maintenance capacity and survival in acid-infused medium. The isolate used in the current study was not subjected to genome recombination procedures to weaken its capacity to tolerate tested organic acids, although its inhibition may not be identical to all Lm isolates, particularly in situations where acid tolerance is increased via prior sublethal organic acid exposure [27].

## 4. Conclusions

FSIS regulations for processing facilities producing RTE deli products require the development of interventions producing* L. monocytogenes* control measures in HACCP, SSOP, and/or other prerequisite programming systems. Organic acid combinations (SL + SDA or SL + PL + SDA) applied in postlethality dips inhibited the growth of* L. monocytogenes* Scott A on RTE deli turkey slices and can be applied as part of a multihurdle food safety system to extend the inhibition the growth of* L. monocytogenes* Scott A in sliced turkey deli products packed and held at refrigeration temperatures. These data can provide supporting information within a* Listeria* control plan for products manufactured under Alternatives 1 or 2b when developing postlethality interventions for processors of aerobically packed RTE deli products.

## Figures and Tables

**Table 1:** Dry bulb temperatures, wet bulb temperatures, time, and smoke setting for each step used in the turkey deli loaf smoke cycle.

Step	Dry bulb temp. (°C)	Wet bulb temp. (°C)	Time (min)	Smoke
1	53.9	−17.8	30	OFF
2	65.6	46.1	120	OFF
3	76.7	58.9	60	OFF
4	85	68.9	Until 71.1°C internal temp	OFF
5	−17.8	−17.8	15 (cold shower)	OFF
